# PARP Inhibitors in Metastatic Prostate Cancer: Bridging Biomarker Complexity and Clinical Decision-Making Through a Pragmatic Treatment Framework

**DOI:** 10.3390/cancers18121949

**Published:** 2026-06-16

**Authors:** Halima Abahssain, Oussama Sabri, Antoine Lemaire, Amine Souadka

**Affiliations:** 1 Department of Oncology, Centre Hospitalier de Valenciennes, 59300 Valenciennes, France; oussamasabri97@gmail.com (O.S.); lemaire-a@ch-valenciennes.fr (A.L.); 2 Equipe de Recherche en Oncologie Translationnelle (EROT), Faculty of Medicine and Pharmacy, Mohammed V University, Rabat 10100, Morocco; 3 Department of Oncology, National Institute of Oncology, Mohammed V University, Rabat 10090, Morocco; 4 Department of Surgery, National Institute of Oncology, Mohammed V University, Rabat 10090, Morocco

**Keywords:** prostate cancer, PARP inhibitors, homologous recombination repair, *BRCA* mutations, precision oncology, Treatment sequencing, biomarkers, castration-resistant prostate cancer

## Abstract

**Background:** The therapeutic landscape of metastatic prostate cancer has rapidly evolved with the integration of biomarker-driven strategies, particularly targeting homologous recombination repair (*HRR*) alterations. Poly(ADP-ribose) polymerase inhibitors (PARPi) have demonstrated clinically meaningful benefit, especially in Breast Cancer genes 1/2-altered tumors, through synthetic lethality. However, the expansion of PARPi across multiple Treatment settings has introduced substantial complexity in patient selection, Treatment sequencing, and biomarker interpretation. This review aims to move beyond a descriptive synthesis of clinical trials and provide a clinically applicable, decision-oriented framework for the use of PARP inhibitors in metastatic prostate cancer. **Methods:** We conducted a narrative review of pivotal phase II and III trials published between 2020 and 2026 evaluating PARPi as monotherapy or in combination strategies. Evidence was critically analyzed with a focus on biomarker relevance, Treatment positioning, and real-world applicability. **Evidence Synthesis:** PARPi consistently improve radiographic progression-free survival, with the most robust and clinically meaningful benefit observed in *BRCA*-altered disease. In contrast, non-*BRCA HRR* alterations demonstrate heterogeneous and often limited predictive value, highlighting the limitations of a binary biomarker approach. Combination strategies in first-line metastatic castration-resistant prostate cancer (mCRPC) have expanded therapeutic options but raise important concerns regarding toxicity, overTreatment, and unclear benefit in biomarker-unselected populations. In parallel, variability in molecular testing strategies and access continues to limit real-world implementation. **Conclusions:** PARP inhibitors represent a cornerstone of precision oncology in metastatic prostate cancer, but their optimal use requires a refined, biomarker-informed approach. In this context, we propose a pragmatic 2026 clinical decision framework integrating molecular characteristics, prior Treatment exposure, and clinical factors. This approach aims to bridge the gap between clinical trial evidence, guideline recommendations, and real-world practice.

## Simple Summary

PARP inhibitors have emerged as a key therapeutic option for metastatic prostate cancer, especially for patients with *BRCA* mutations, who have the most significant clinical benefit. However, responses are more heterogeneous in patients with non-*BRCA* alterations, highlighting the importance of accurate biomarker selection. Combination strategies associating PARP inhibitors and androgen receptor pathway inhibitors have shown promising results and strengthened the role of these therapies earlier in the disease evolution. Despite these advances, many challenges remain regarding patient selection, Treatment sequencing, toxicity management, and access to molecular testing in routine practice. This review aims to provide a practical and clinically relevant overview of PARP inhibitor use in metastatic prostate cancer by integrating current biological insights, major clinical trial results, and real-world considerations to support personalized Treatment decision-making.

## 1. Introduction

The therapeutic landscape of metastatic prostate cancer has undergone a profound transformation with the integration of precision oncology. Among the most practice-changing advances is the identification of alterations in homologous recombination repair (*HRR*) genes, particularly Breast Cancer gene 1 (*BRCA1*) and *BRCA*2, which define a biologically distinct subgroup with both prognostic and therapeutic implications [1,2].

These defects create a therapeutically exploitable vulnerability through the principle of synthetic lethality, positioning Poly(ADP-ribose) polymerase inhibitors (PARPi) as a cornerstone of targeted therapy in advanced disease [3,4].

Randomized phase III trials have established PARP inhibition as an effective Treatment strategy in metastatic castration-resistant prostate cancer (mCRPC), initially as biomarker-selected monotherapy and, more recently, in combination with androgen receptor pathway inhibitors (ARPIs) [5,6,7].

However, the expansion of PARPi across multiple therapeutic settings has introduced substantial clinical complexity. Key questions remain regarding patient selection, optimal timing of therapy, and Treatment sequencing, particularly given the biological heterogeneity of *HRR* alterations and the variability in molecular testing strategies [8,9].

In this context, this review aims to provide a pragmatic and clinically applicable framework for the use of PARP inhibitors in metastatic prostate cancer, integrating biological rationale, critical interpretation of pivotal trials, and real-world considerations. We propose a 2026 decision-oriented approach to support biomarker-driven Treatment strategies in routine clinical practice.

## 2. Why Now (2020–2026)

Over the past decade, the management of metastatic prostate cancer has transitioned from a largely sequence-based approach to a biomarker-driven strategy. Between 2020 and 2026, several pivotal phase III trials established PARP inhibitors as an effective therapeutic option, initially in biomarker-selected mCRPC and more recently in earlier Treatment settings through combination strategies [5,7,10,11].

This expansion across the disease continuum has fundamentally reshaped clinical decision-making. The challenge is no longer limited to identifying eligible patients, but now includes determining the optimal timing, sequencing, and integration of PARP inhibitors within an increasingly complex therapeutic landscape.

At the same time, the implementation of biomarker-driven strategies remains heterogeneous. Variability in access to molecular testing and differences in clinical workflows continue to create a gap between trial evidence and real-world practice [12].

In this context, there is a clear need to move beyond trial-by-trial interpretation and toward a structured, clinically applicable framework to guide the use of PARP inhibitors in routine practice.

## 3. Clinically Relevant Biology: Beyond HRR Status

### 3.1. HRR Deficiency and Synthetic Lethality

*HRR* is a critical DNA repair pathway that ensures genomic stability through accurate repair of double-strand breaks. Its alteration plays a central role in prostate cancer biology. Among *HRR* genes, *BRCA*2, and to a lesser extent *BRCA*1, are key tumor suppressors, with *BRCA*2 representing the most frequent and clinically relevant alteration in metastatic disease [9,13].

A meta-analysis of metastatic prostate cancer cohorts reported *BRCA*2 mutations in 10.26% and 4.51% of patients at the somatic and germline levels, respectively, whereas *BRCA*1 mutations were less frequent, occurring in 1.1% and 0.94% of patients as somatic and germline alterations, respectively [14].

PARPi-mediated synthetic lethality operates through two complementary mechanisms. First, catalytic inhibition of PARP1/2 prevents the repair of single-strand DNA breaks (SSBs), leading to their conversion into double-strand breaks (DSBs) at stalled replication forks. Second, PARP inhibitors trap PARP–DNA complexes at sites of damage, generating cytotoxic protein–DNA adducts that are far more lethal than unrepaired SSBs alone. In *HRR*-proficient cells, these DSBs are efficiently resolved through homologous recombination. In contrast, *BRCA*1/2-deficient tumor cells lack functional *HRR* capacity and are forced to rely on alternative repair pathways, ultimately resulting in genomic instability and cell death. This mechanistic duality explains both the potency of PARPi in *BRCA*-altered disease and the more limited activity observed in tumors with non-*BRCA HRR* alterations, where residual repair capacity counterbalances the cytotoxic effect [15,16].

Loss of *BRCA* function results in defective homologous recombination, forcing tumor cells to rely on alternative DNA repair mechanisms mediated by PARP enzymes. Pharmacologic inhibition of PARP in this context leads to the accumulation of DNA damage and tumor cell death, a phenomenon known as synthetic lethality [3]. Clinically, PARP inhibitor efficacy is largely driven by *BRCA*2-altered tumors, which demonstrate higher response rates and improved survival outcomes compared with other *HRR* alterations [5,17].

### 3.2. HRR Heterogeneity: Beyond a Binary Biomarker

The concept of “*BRCA*ness” has been proposed to extend PARP inhibitor sensitivity beyond *BRCA*1/2-mutated tumors. However, in prostate cancer, this concept masks substantial biological heterogeneity [18,19,20].

Non-*BRCA HRR* alterations, including ATM, *CDK12*, and CHEK2, differ significantly in their functional role within DNA repair pathways. While *BRCA*1/2 directly mediate homologous recombination, *ATM* and *CHEK2* act primarily as DNA damage sensors, and *CDK12* regulates transcription of DNA repair genes. As a result, these alterations do not uniformly confer true homologous recombination deficiency [18,21,22].

**Figure 1:**
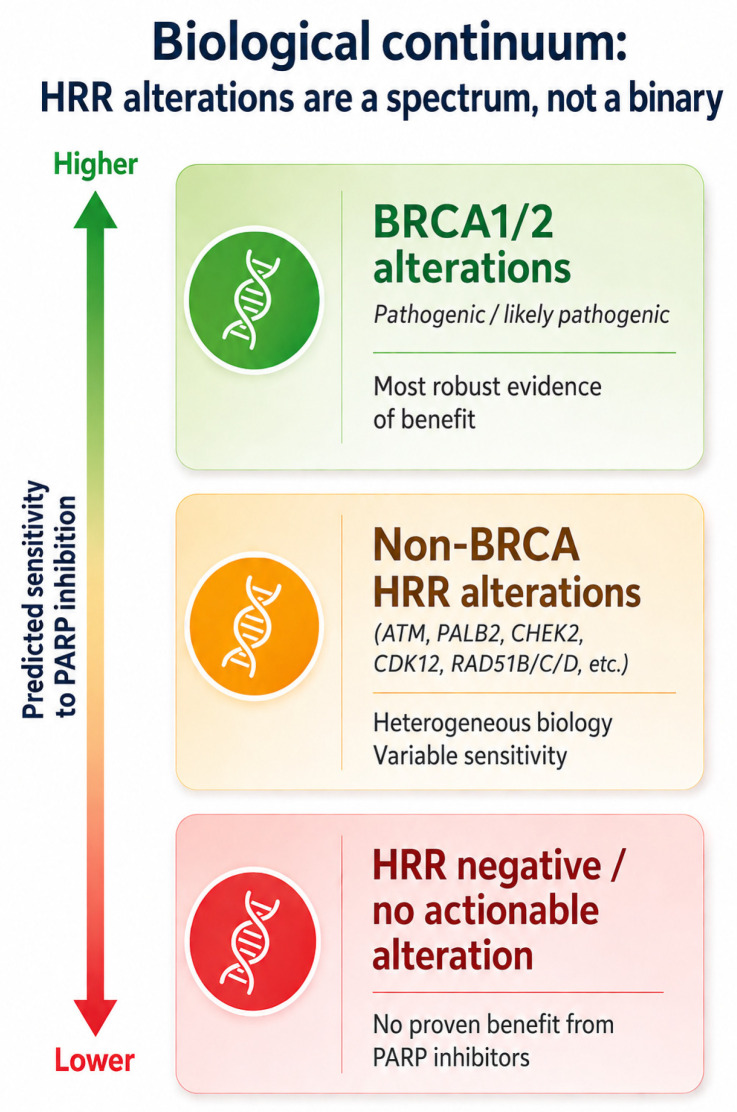
Biological continuum of homologous recombination repair alterations and predicted sensitivity to PARP inhibition in metastatic prostate cancer.

This biological diversity translates into heterogeneous clinical outcomes. Across prospective studies, responses to PARP inhibitors are consistently lower and less predictable in non-*BRCA HRR* subgroups, supporting the concept that *HRR* alterations represent a functional spectrum rather than a binary predictive biomarker [17,18] (Figure 1).

### 3.3. Rationale for Combination Strategies

**Androgen Receptor (AR)—PARP Crosstalk**

DNA repair mechanisms and AR signaling interact in both directions in prostate cancer. By attaching to androgen response elements in their regulatory areas, AR signaling directly controls the transcriptional expression of important *HRR* genes, such as *RAD51*. Preclinical research has shown that AR enhances homologous recombination and efficiently accumulates *RAD51* foci at DNA damage sites, whereas AR knockdown impairs *HRR* capacity and increases sensitivity to DNA-damaging chemicals [23]. Consequently, a state of functional *HRR* deficiency is induced by pharmacological inhibition of AR signaling, which offers a mechanistic explanation for PARP inhibitor sensitization independent of underlying *HRR* gene alterations.

On the other hand, AR transcriptional activity is directly influenced by PARP enzymes. AR is recruited to the promoters of its target genes by PARP1, a transcriptional co-activator of AR. Through FOXA1-dependent chromatin remodeling, PARP2 plays a more specific role in AR-mediated transcription. Pharmacological inhibition of PARP2 reduces AR target gene expression to a level similar to that of enzalutamide therapy.

Taken together, this bidirectional crosstalk provides the mechanistic basis for the therapeutic rationale of combining AR pathway inhibitors with PARP inhibitors in first-line mCRPC, while also highlighting that clinical benefit in *HRR*-proficient populations may depend on both the ARPi responsiveness of tumor cells and the PARP-trapping potency of the selected PARPi [24,25].

This rationale supports the design of phase III trials evaluating PARP inhibitor combinations in first-line mCRPC. However, clinical benefit appears to remain greatest in *HRR*-altered tumors, particularly those harboring *BRCA*1/2 mutations, while efficacy in *HRR*-proficient populations is more modest and remains debated.

**Radiation Therapy and PARP Inhibitor Combinations**

Combining PARP inhibitors with radiation-based approaches is an area of special biological and therapeutic interest. Both external beam radiotherapy and radioligand therapy (RLT) primarily induce DNA single-strand breaks (SSBs), which are repaired via PARP-dependent base excision repair [26].

PARP inhibition converts these SSBs into lethal double-strand breaks, amplifying cytotoxicity through a radiosensitizing mechanism that operates independently of *HRR* status. In the context of ^177^Lu-PSMA-617-based RLT, a standard of care in mCRPC, this provides an exciting background for combination with PARPi.

The LuPARP phase 1 trial, enrolling patients with PSMA-avid mCRPC, has demonstrated the feasibility and preliminary activity of combining ^177^Lu-PSMA-617 with olaparib, with an acceptable safety profile and encouraging PSA response rates across dose escalation cohorts [27].

Randomized clinical trials are needed to confirm efficacy and better select patients who benefit from the association. Nevertheless, PARPi combined with RLT seems to be one of the most mechanistically coherent and clinically actionable future strategies in this disease.

## 4. Misconceptions

Despite the rapid integration of PARP inhibitors into clinical practice, several misconceptions continue to influence Treatment decisions.

First, *HRR* alterations are frequently interpreted as a binary predictive biomarker of response to PARP inhibition. However, both biological and clinical evidence indicate that *HRR* alterations represent a heterogeneous spectrum, with *BRCA*2 consistently associated with the most robust clinical benefit, whereas alterations in genes such as *ATM*, *CDK12*, and *CHEK2* are associated with limited or inconsistent responses [5,18].

Second, there is a tendency to group all *HRR* alterations together despite clear functional differences. This oversimplification may lead to an overestimation of Treatment benefit in non-*BRCA* molecular subgroups and highlights the need for a more gene-specific interpretation of *HRR* status [18,28].

Third, positive results from combination trials in biomarker-unselected populations may be overinterpreted. While studies such as PROpel demonstrated improved radiographic progression-free survival, the absence of consistent overall survival benefit and the negative *HRR*-negative cohort in MAGNITUDE argue against assuming uniform efficacy across all patients [10,11].

Fourth, the assumption that earlier use of PARP inhibitors is inherently superior remains unproven. Although combination strategies improve disease control, they are associated with increased toxicity and may expose patients with uncertain benefit to unnecessary Treatment [29,30].

The Phase II BRCAAway trial provides important but preliminary insights by suggesting that upfront combination therapy may prolong progression-free survival compared with sequential strategies in patients with *HRR*-mutated mCRPC. However, the limited sample size, selected population, and absence of mature survival data preclude definitive conclusions regarding the superiority of combination over an optimized, biomarker-driven sequencing approach [31].

Finally, molecular testing is often perceived as straightforward, whereas in practice it is subject to significant variability. Differences in testing platforms, tissue availability, and interpretation of genomic alterations may result in inconsistent patient selection across institutions [4,32].

Taken together, these misconceptions highlight that PARP inhibitors cannot be used based on simplified assumptions and require a structured, biomarker-driven approach.

## 5. Pivotal Trials and Clinical Indications of PARP Inhibitors in Prostate Cancer

The clinical development of PARP inhibitors in prostate cancer has been driven by a series of pivotal trials conducted predominantly in metastatic castration-resistant prostate cancer (mCRPC). These studies established *HRR* alterations, particularly *BRCA*1/2, as predictive biomarkers of response and led to the approval of multiple PARP inhibitor-based strategies.

### 5.1. PARP Inhibitor Monotherapy: Proof of Principle in Biomarker-Selected Disease

The clinical development of PARP inhibitors in prostate cancer was initially grounded in a biomarker-selected strategy targeting tumors with homologous recombination repair (*HRR*) deficiency, particularly *BRCA*1/2 alterations. Early phase II studies, such as TOPARP-A, provided the first clinical evidence that PARP inhibition could induce meaningful and durable responses in patients with DNA repair-deficient tumors, thereby validating the concept of synthetic lethality in prostate cancer [33].

This approach was subsequently confirmed in the phase III PROfound trial, which demonstrated that olaparib significantly improved radiographic progression-free survival compared with physician’s choice of AR pathway inhibitors in men with *HRR*-mutated mCRPC progressing after ARPI exposure [5]. Importantly, subgroup analyses showed that this benefit was largely driven by *BRCA*1/2-altered tumors, whereas activity in other *HRR* subgroups was more limited.

Consistent findings were observed with rucaparib in TRITON2 and TRITON3. In TRITON3, a clear improvement in progression-free survival was demonstrated in the *BRCA* subgroup, while no significant benefit was observed in *ATM*-altered tumors, further emphasizing the heterogeneity of *HRR* alterations and their differential predictive value [6,17] (Table 1).

Taken together, these data establish PARP inhibitor monotherapy as a biomarker-driven Treatment strategy, with the most consistent and clinically meaningful benefit observed in *BRCA*-altered disease.

### 5.2. Combination Strategies: Earlier Use, Broader Populations, Greater Uncertainty

More recently, PARP inhibitors have been evaluated in combination with ARPIs in earlier Treatment settings, based on the biological rationale that AR inhibition may induce a state of functional homologous recombination deficiency.

Three pivotal phase III trials—PROpel, MAGNITUDE, and TALAPRO-2—have demonstrated significant improvements in radiographic progression-free survival with PARP inhibitor-based combinations in first-line mCRPC, although important differences in trial design and patient selection limit direct comparisons [10,29,34].

PROpel evaluated olaparib plus abiraterone in an unselected population and demonstrated a significant rPFS benefit, raising the possibility of broader use beyond biomarker-selected patients. In contrast, MAGNITUDE prospectively stratified patients according to *HRR* status and discontinued enrollment in the *HRR*-negative cohort due to lack of benefit, providing strong evidence that biomarker selection remains critical. TALAPRO-2 further demonstrated improvements in both rPFS and overall survival with talazoparib plus enzalutamide, although interpretation is influenced by the inclusion of both unselected and *HRR*-enriched populations (Table 1).

Overall, these trials indicate that combination strategies can enhance disease control when used earlier in the disease course. However, the magnitude of benefit appears heterogeneous and is largely driven by *HRR*-positive, particularly *BRCA*-altered tumors, raising important questions regarding patient selection and the risk–benefit balance in biomarker-unselected populations.

## 6. Cross-Trial Differences: Why These Studies Are Not Interchangeable

A major challenge in interpreting the PARP inhibitor landscape lies in the tendency to compare results across trials as if they were directly interchangeable. In reality, substantial heterogeneity exists in study design, patient populations, biomarker definitions, and prior Treatments.

PROpel evaluated an unselected population, whereas MAGNITUDE incorporated prospective *HRR* stratification and discontinued enrollment in *HRR*-negative patients due to lack of benefit [10,34]. TALAPRO-2 included both unselected and *HRR*-enriched cohorts, further complicating interpretation [29].

In addition, differences in control arms, endpoint definitions, and prior exposure to AR pathway inhibitors limit the validity of indirect comparisons. Apparent differences in efficacy may therefore reflect underlying population heterogeneity rather than intrinsic differences between Treatment strategies.

These limitations highlight the need for caution when interpreting cross-trial data and reinforce the importance of biomarker-driven Treatment decisions.

## 7. Treatment Sequencing in 2026

### 7.1. Timing Is Strategy

The integration of PARP inhibitors into earlier lines of therapy has shifted the clinical question from identifying eligible patients to determining the optimal timing of treatment initiation.

**Figure 2:**
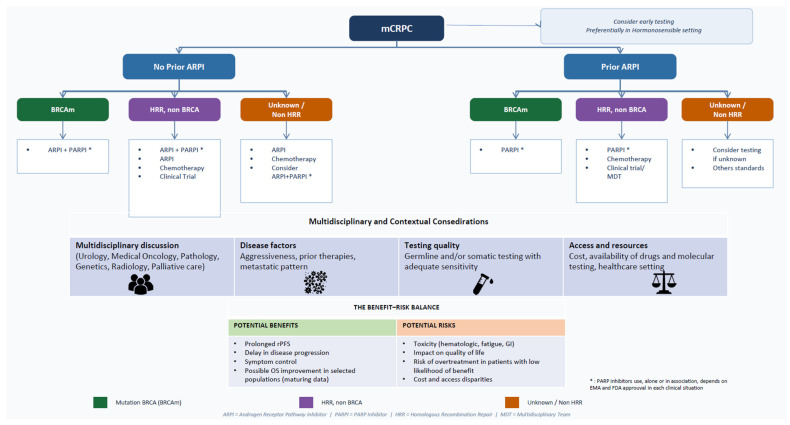
Proposed 2026 clinical framework for PARP inhibitor integration and Treatment sequencing in metastatic castration-resistant prostate cancer.

Two distinct strategies have emerged. The first is early intensification, in which PARP inhibitors are combined with AR pathway inhibitors in first-line mCRPC to maximize initial disease control. The second is a delayed, biomarker-driven approach, reserving PARP inhibitor monotherapy for patients with confirmed *HRR* alterations, particularly *BRCA*1/2, after progression on AR-targeted therapy (Figure 2).

These strategies reflect different therapeutic philosophies rather than equivalent standards of care and should not be considered interchangeable.

### 7.2. Sequencing Challenges in Clinical Practice

Several unresolved questions continue to complicate treatment sequencing. It remains unclear whether early exposure to PARP inhibitors affects the efficacy of subsequent therapies or whether delaying their use may result in missed opportunities in aggressive disease. In addition, prior treatments, including AR pathway inhibitors and chemotherapy, may influence PARP inhibitor sensitivity. Emerging resistance mechanisms, such as *BRCA* reversion mutations, further complicate long-term Treatment strategies.

In the absence of prospective sequencing trials, clinical decisions must rely on indirect evidence and individualized assessment (Figure 2).

The proposed algorithm should be viewed as a pragmatic, decision-support framework derived from currently available evidence, rather than a formal guideline, and is intended to reflect both established indications and persistent areas of uncertainty.

### 7.3. Biomarker-Centered Approach

In practice, Treatment sequencing should be guided by an integrated evaluation of molecular and clinical factors. Patients with *BRCA*-altered disease represent the most appropriate candidates for PARP inhibitor-based strategies, given the consistent evidence of benefit. In contrast, a more cautious approach is warranted in non-*BRCA HRR* alterations due to the heterogeneity of responses, with prioritization of clinical trials when available. In *HRR*-negative disease, the rationale for PARP inhibitor use remains limited outside selected contexts.

Patient-related factors, including performance status, comorbidities, disease burden, prior therapies, and Treatment goals, are essential in tailoring Treatment strategies.

## 8. Tolerability and Patient-Reported Outcomes: The Hidden Trade-Off

The tolerability profile of PARP inhibitors in prostate cancer is largely driven by hematologic toxicity, with clinically meaningful differences observed between biomarker-selected monotherapy and combination strategies with androgen receptor pathway inhibitors (ARPIs). Across trials, anemia represents the most consistent and clinically relevant class-defining adverse event.

In biomarker-selected settings, PARP inhibitor monotherapy is generally associated with a manageable safety profile. In PROfound, olaparib was primarily associated with anemia and nausea, with relatively limited Treatment discontinuation in previously treated *HRR*-altered mCRPC [5].

In contrast, combination strategies are consistently associated with increased hematologic burden. In PROpel, the addition of olaparib to abiraterone resulted in higher rates of grade ≥ 3 anemia and more frequent Treatment interruptions, while in TALAPRO-2, talazoparib plus enzalutamide led to substantial myelosuppression, with anemia as the predominant severe toxicity [38,39].

Importantly, toxicity profiles also vary across agents. Thrombocytopenia is more frequently observed with niraparib- and talazoparib-based regimens, as illustrated in MAGNITUDE and TALAPRO-2, whereas it is less prominent with olaparib monotherapy [11].

Fatigue is common across all strategies but is typically low-grade and rarely dose-limiting, although its cumulative impact should not be underestimated in patients receiving prolonged Treatment in earlier disease settings [5,40].

Beyond drug-specific effects, tolerability should be understood as a strategy-dependent trade-off. Monotherapy offers a more favorable safety profile and simpler management, whereas combination approaches provide earlier disease control at the cost of increased toxicity, more frequent dose modifications, and greater supportive-care requirements. This balance becomes particularly relevant as PARP inhibitors move into earlier disease settings, where patients are less symptomatic and long-term tolerability is a key consideration, raising concerns about potential overTreatment in biomarker-unselected populations.

Dose adaptation is therefore an integral component of PARP inhibitor management rather than a marker of Treatment failure. Across trials, including dedicated analyses from PROpel, most adverse events were effectively managed through supportive care, Treatment interruptions, and stepwise dose reductions, allowing continued Treatment exposure [38].

In clinical practice, maintaining patients on therapy often depends more on proactive toxicity management than on strict adherence to initial dosing.

Despite the increased hematologic burden associated with combination strategies, patient-reported outcomes (PROs) have been consistently reassuring. Global health-related quality of life (HRQoL) has been preserved across both monotherapy and combination settings. In PROfound, olaparib was associated with improved pain control and better-preserved HRQoL compared with ARPI after progression [41].

Similarly, in PROpel, MAGNITUDE, and TALAPRO-2, PARP inhibitor-based combinations delayed deterioration in multiple patient-reported domains without clinically meaningful worsening of overall quality of life [42,43,44].

Taken together, these findings indicate that, when appropriately managed, the toxicity profile of PARP inhibitors does not negate their clinical benefit. However, tolerability must be considered a central component of Treatment strategy, particularly when selecting between early combination approaches and biomarker-driven monotherapy (Table 2).

## 9. Real-World Implementation: Bridging the Gap Between Evidence and Practice

Despite the rapid integration of PARP inhibitor-based strategies into clinical guidelines, their real-world implementation remains heterogeneous. The translation of trial evidence into routine practice is challenged by structural, logistical, and biological factors that extend beyond clinical trial settings [12,32].

### 9.1. Access to Molecular Testing

Systematic testing for *HRR* alterations is a prerequisite for optimal patient selection, yet access to molecular diagnostics varies across healthcare systems. Limitations related to next-generation sequencing (NGS) availability, reimbursement policies, tissue acquisition, and turnaround times may delay or restrict access to genomic profiling [32,47,48].

However, access to testing should be balanced against the expected benefit of PARP inhibitor therapy. Indeed, the prevalence of *BRCA* mutations in advanced prostate cancer is approximately 10%, meaning that only a relatively small proportion of patients are ultimately eligible for these Treatments [14]. Moreover, a clear overall survival benefit has been primarily demonstrated with the combination of a PARP inhibitor and an ARPI in patients with *BRCA* alterations, as observed in the TALAPRO-2 study [29]. In contrast, several other studies evaluating PARP inhibitors alone or in combination in the castration-resistant setting have mainly reported improvements in radiographic progression-free survival rather than overall survival.

This issue may become even more relevant as ARPIs are increasingly used in the metastatic hormone-sensitive setting, potentially reducing the number of patients who subsequently reach Treatment scenarios similar to those evaluated in mCRPC trials. Therefore, access to broad molecular testing should be carefully weighed against the expected clinical benefit and the feasibility of accessing the corresponding targeted therapies.

### 9.2. Interpretation Challenges

Even when testing is available, interpretation remains complex. Variants of uncertain significance, allelic status, and differences in gene panels can influence clinical decision-making. Current gene-based classifications do not fully capture functional homologous recombination deficiency, particularly in non-*BRCA* alterations [4,32].

### 9.3. Timing and Integration into Care Pathways

Ideally, *HRR* testing should be performed early in the disease course to guide Treatment strategy. However, in routine practice, testing is often delayed, limiting the ability to implement biomarker-driven approaches in a timely manner [12].

### 9.4. Health System Variability and Access to Treatment

Access to PARP inhibitors may also vary depending on regulatory approvals, reimbursement policies, and local infrastructure, further widening the gap between clinical trial evidence and real-world practice.

Both the U.S. Food and Drug Administration (FDA) and the European Medicines Agency (EMA) recognize PARP inhibitors as an established Treatment option in metastatic castration-resistant prostate cancer (mCRPC), although approved indications differ according to biomarker status and Treatment combinations. Consequently, Treatment eligibility depends not only on molecular profiling but also on regional reimbursement policies and approved indications, which may contribute to variability in real-world access to PARP inhibitor–based therapies (Table 3).

## 10. Future Directions

**Figure 3:**
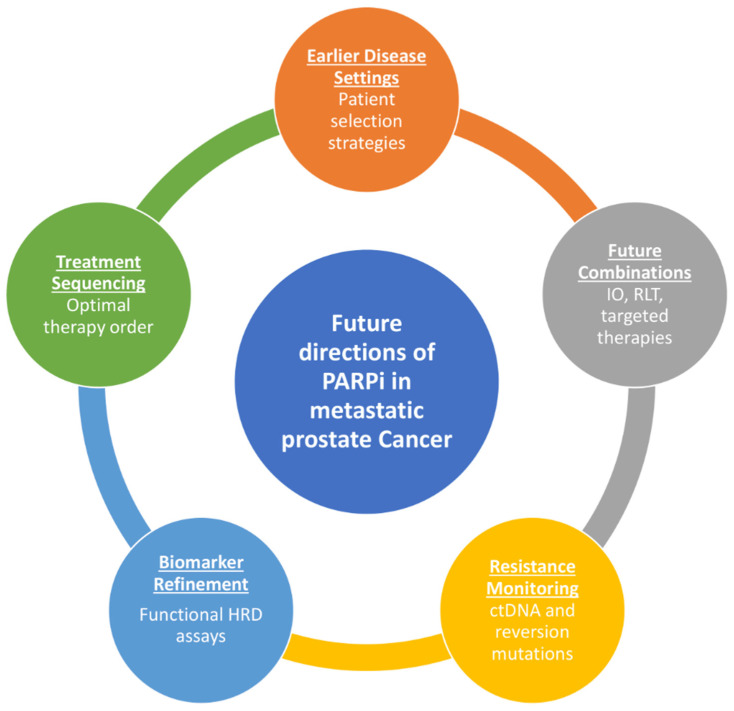
Future directions of PARP inhibitor development in metastatic prostate cancer.

Future clinical development of PARP inhibitors in prostate cancer is increasingly shifting from expansion of indications toward refinement of patient selection and optimization of therapeutic strategies. While PARP inhibitor-based approaches are now established across multiple disease settings, several critical gaps remain that will define the next phase of development (Figure 3).

### 10.1. Refining Biomarker Selection: Beyond Gene-Based HRR Classification

A major limitation of current clinical practice is the reliance on gene-based *HRR* classification as the primary biomarker for PARP inhibitor sensitivity. While *BRCA*1/2 alterations, particularly *BRCA*2, consistently predict clinical benefit, the predictive value of non-*BRCA HRR* alterations remains heterogeneous and often limited.

This has driven increasing interest in functional biomarkers of homologous recombination deficiency (HRD), including genomic scar assays, mutational signatures, and composite genomic classifiers. These approaches aim to capture the biological consequences of DNA repair deficiency rather than the presence of individual gene alterations. However, these tools remain insufficiently standardized and lack prospective validation in prostate cancer, limiting their integration into routine clinical practice.

The development of prostate-specific HRD frameworks represents a key research priority, as current assays have largely been extrapolated from breast and ovarian cancers and may not adequately reflect the genomic architecture of prostate tumors.

### 10.2. Treatment Sequencing: Defining an Evidence-Based Strategy

A major unresolved challenge in the PARP inhibitor landscape is the absence of prospective data defining optimal Treatment sequencing. Current strategies are largely derived from indirect cross-trial comparisons across heterogeneous populations and study designs, limiting their clinical interpretability. Critical questions remain, including whether early use of PARP inhibitors in combination strategies alters the efficacy of subsequent therapies, whether delayed introduction may compromise outcomes in biologically aggressive disease, and how prior exposure to androgen receptor pathway inhibitors, chemotherapy, or radioligand therapy modulates PARP inhibitor sensitivity. In this context, defining an evidence-based sequencing strategy represents a key priority for the field and will require dedicated prospective trials supported by high-quality, real-world longitudinal data.

### 10.3. Resistance Mechanisms and Dynamic Biomarkers

Acquired resistance to PARP inhibitors is emerging as a central clinical challenge. Among the most clinically relevant mechanisms are *BRCA* reversion mutations, which restore homologous recombination function and are associated with reduced Treatment efficacy.

Importantly, emerging evidence suggests that such reversion events may occur even prior to PARP inhibitor exposure, potentially as a consequence of prior DNA-damaging therapies. This observation has important implications for Treatment sequencing and highlights the need for dynamic molecular monitoring.

Liquid biopsy approaches, particularly circulating tumor DNA (ctDNA), offer a promising strategy to detect resistance mechanisms in real time and may allow adaptive Treatment strategies. However, their clinical utility in guiding PARP inhibitor therapy remains to be prospectively validated.

### 10.4. Earlier Disease Settings: Intensification Versus Patient Selection

The integration of PARP inhibitors into metastatic castration-sensitive prostate cancer (mCSPC) has recently gained strong support from phase III trials. In TALAPRO-3, talazoparib plus enzalutamide significantly improved radiographic progression-free survival in patients with *HRR*-altered mCSPC (HR 0.48), with benefit observed in both *BRCA*- and non-*BRCA*-altered tumors [37].

However, Treatment intensification must be balanced against increased toxicity, particularly hematologic adverse events. Therefore, the challenge is no longer whether PARP inhibitors should be used in mCSPC, but rather identifying which patients derive sufficient benefit to justify upfront combination therapy versus a sequential, biomarker-driven approach.

### 10.5. Future Therapeutic Strategies: Combination and Beyond

Beyond current doublet strategies, future development is likely to explore more complex combinations, including PARP inhibitors with immunotherapy, radioligand therapy, or other targeted agents. However, early data suggest that these approaches will require biomarker enrichment and should not be generalized across unselected populations.

Ultimately, progress in this field will depend on integrating biological understanding with clinical trial design. The next generation of studies will need to move beyond demonstrating efficacy toward defining the right patient population, the optimal timing, and the appropriate sequencing of therapies in a biologically rational manner.

## 11. Conclusions

The integration of PARP inhibitors into the therapeutic landscape of metastatic prostate cancer represents a major advance in precision oncology. However, their expanding use has shifted the clinical challenge from demonstrating efficacy to optimizing their integration within complex Treatment pathways.

A consistent message emerges across studies: *HRR* alterations are not interchangeable. Clinical benefit from PARP inhibition is predominantly driven by *BRCA*, whereas non-*BRCA* alterations confer variable and frequently limited therapeutic benefit.

The emergence of combination strategies introduces a new therapeutic paradigm, balancing earlier disease control against increased toxicity and the risk of overTreatment. In this context, Treatment decisions must integrate molecular characteristics, clinical factors, and patient preferences.

Bridging the gap between clinical trial evidence and real-world implementation remains essential. Future progress will depend on improved biomarker definition, a better understanding of resistance mechanisms, and the generation of comparative data to guide sequencing strategies.

Ultimately, the future of PARP inhibitor therapy will depend less on expanding indications than on achieving true biological precision through more selective and individualized Treatment strategies.

## Figures and Tables

**Table 1:** Pivotal trials of PARP inhibitors in prostate cancer.

Trial	Drug/Combination	Setting	Biomarker Required	Line of Therapy	Comparator	Primary Endpoint	Key Result	Dominant Toxicity
**PROfound** [5]	Olaparib monotherapy	mCRPC	*HRR* gene alterations	Post-progression on enzalutamide or abiraterone	Physician’s choice (Enzalutamide or Abiraterone)	ibPFS (Cohort A)	Median ibPFS 7.4 mo vs. 3.6 mo in Cohort A (HR 0.34, *p* < 0.001)	Anemia (46%), nausea (41%)
**TRITON3** [6]	Rucaparib monotherapy	mCRPC	Deleterious *BRCA*1/2 and *ATM* gene alterations	Post-progression on second-generation androgen receptor pathway inhibitors (ARPI) such as abiraterone and enzalutamide	Physician’s choice among docetaxel, enzalutamide, or abiraterone	Imaging-based progression-free survival (PFS)	Median imaging-based PFS in the *BRCA* subgroup (11.2 months vs. 6.4 months; HR = 0.50, *p* < 0.001) No significant difference in the imaging-based PFS in the *ATM* subgroup	Fatigue, nausea, and anemia Grade 3 complications were slightly more common in the rucaparib group (60% vs. 53%)
**MAGNITUDE** [34]	Niraparib + Abiraterone (AAP)	mCRPC	*HRR*+ alterations, specifically *BRCA*1/2	First-line mCRPC	Placebo + AAP	rPFS	Significant rPFS benefit (HR 0.53 in *BRCA*1/2; HR 0.73 in overall *HRR*+)	Anemia (52.4%), hypertension (34%), thrombocytopenia (24.1%)
**PROpel** [10]	Olaparib + Abiraterone	mCRPC	Unselected	First-line mCRPC	Placebo + Abiraterone	ibPFS (Investigator-assessed)	Median ibPFS 24.8 mo vs. 16.6 mo (HR 0.66, *p* < 0.001)	Anemia (46%), fatigue (37.2%), nausea (28.1%)
**TALAPRO-2** [29]	Talazoparib + Enzalutamide	mCRPC	Unselected for main cohort	First-line mCRPC	Placebo + Enzalutamide	rPFS (by BICR)	Significantly improved OS (HR 0.80) and rPFS (HR 0.67)	Anemia (68%), neutropenia (38%), fatigue (35%)
**AMPLITUDE** [35]	Niraparib + Abiraterone	mCSPC	*HRR*+	First-line	Placebo + AAP	rPFS	Improved rPFS in *HRR*-mutated population (HR ~0.63)	Anemia, hypertension
**TALAPRO-3** [36,37]	Talazoparib + Enzalutamide	mCSPC	*HRR*+	First-line	Placebo + Enzalutamide	rPFS	Improved rPFS (77% vs. 56%) HR 0.48; *p* < 0.001	Hematologic toxicity, particularly grade ≥3 anemia (51%), neutropenia, thrombocytopenia; transfusions in 40% of patients

Abbreviations: AAP, abiraterone acetate plus prednisone; mCRPC, metastatic castration-resistant prostate cancer; mCSPC, metastatic castration-sensitive prostate cancer; *HRR*, homologous recombination repair; ARPI, androgen receptor pathway inhibitor; rPFS, radiographic progression-free survival; OS, overall survival.

**Table 2:** Comparative safety profiles across major PARP inhibitor trials in metastatic castration-resistant prostate cancer.

Adverse Event/Outcome	PROfound Olaparib Monotherapy [5]	PROpel Olaparib + Abiraterone [10]	TALAPRO-2 Talazoparib + Enzalutamide [7]	MAGNITUDE Niraparib + AAP [45]
Anemia, all grades	46%	46%	66%	50%
Anemia, grade ≥ 3	21%	15.1%	46%	30.2%
Fatigue, all grades	41%	37.2%	34%	29.7%
Fatigue, grade ≥ 3	3%	2.3%	4%	3.8%
Nausea, all grades	41%	28.1%	21%	24.5%
Nausea, grade ≥ 3	1%	0.3%	<1%	0.5%
Neutropenia, all grades	34% [46]	23% [46]	36%	15.1%
Neutropenia, grade ≥ 3	3% [46]	5% [46]	18%	5.2%
Thrombocytopenia, grade ≥ 3	4% [46]	1.2% [46]	7%	3.8%
Grade ≥ 3 adverse events (overall)	51%	47.2%	75%	72.2%
Dose reduction	22%	20.1%	53%	20.3%
Treatment interruption	45%	44.7%	62%	49.1%
Treatment discontinuation due to adverse events	18%	13.8%	19%	15.1%

**Table 3:** Current FDA and EMA approvals of PARP inhibitor-based strategies in metastatic prostate cancer.

PARP Inhibitor Strategy	FDA-Approved Indication	EMA-Approved Indication	Key Supporting Trial
Olaparib monotherapy	Adult patients with deleterious or suspected deleterious germline or somatic *HRR* gene-mutated mCRPC who have progressed following prior Treatment with enzalutamide or abiraterone [49]	Adult patients with *BRCA*1/2-mutated mCRPC, germline and/or somatic, who have progressed following prior therapy that included a new hormonal agent. [50]	PROfound
Olaparib + abiraterone + prednisone/prednisolone	Adult patients with deleterious or suspected deleterious *BRCA*-mutated mCRPC, as determined by an FDA-approved companion diagnostic test [51]	Adult patients with mCRPC in whom chemotherapy is not clinically indicated. No mandatory genomic testing required according to the EMA label [50]	PROpel
Talazoparib + enzalutamide	Adult patients with *HRR* gene-mutated mCRPC [52]	Adult patients with mCRPC in whom chemotherapy is not clinically indicated [53]	TALAPRO-2
Niraparib + abiraterone + prednisone/prednisolone	Adult patients with deleterious or suspected deleterious *BRCA*-mutated mCRPC, as determined by an FDA-approved test [54]	Adult patients with mCRPC and *BRCA*1/2 mutations, germline and/or somatic, in whom chemotherapy is not clinically indicated [55]	MAGNITUDE
Niraparib + abiraterone + prednisone + ADT	Adult patients with deleterious or suspected deleterious *BRCA*2-mutated mCSPC [56]	Adult patients with mCSPC and *BRCA*1/2 mutations, germline and/or somatic, in combination with androgen deprivation therapy [55]	AMPLITUDE
Rucaparib monotherapy	Adult patients with deleterious *BRCA*-mutated mCRPC, germline and/or somatic, previously treated with androgen receptor-directed therapy; patients should be selected using an FDA-approved companion diagnostic [57]	No EMA-approved prostate cancer indication	TRITON2/TRITON3

Abbreviations: ADT, androgen deprivation therapy; EMA, European Medicines Agency; FDA, U.S. Food and Drug Administration; *HRR*, homologous recombination repair; mCRPC, metastatic castration-resistant prostate cancer; mCSPC, metastatic castration-sensitive prostate cancer; PARP, poly(ADP-ribose) polymerase. Important: Regulatory indications may evolve over time and may differ according to regional implementation, reimbursement policies, and future label updates.

## Data Availability

No new data were generated or analyzed in this study.

## Abbreviations

The following abbreviations are used in this manuscript:

ADT	Androgen deprivation therapy
AAP	Abiraterone acetate plus prednisone
AR	Androgen receptor
ARPI	Androgen receptor pathway inhibitor
*ATM*	Ataxia telangiectasia mutated
BICR	Blinded independent central review
*BRCA*	Breast cancer gene
ctDNA	Circulating tumor DNA
DNA	Deoxyribonucleic acid
DSB	Double-strand break
EMA	European Medicines Agency
FDA, U.S.	Food and Drug Administration
HRD	Homologous recombination deficiency
*HRR*	Homologous recombination repair
HRQoL	Health-related quality of life
ibPFS	Imaging-based progression-free survival
mCRPC	Metastatic castration-resistant prostate cancer
mCSPC	Metastatic castration-sensitive prostate cancer
NGS	Next-generation sequencing
OS	Overall survival
PARP	Poly(ADP-ribose) polymerase
PARPi	Poly(ADP-ribose) polymerase inhibitor
PFS	Progression-free survival
PRO	Patient-reported outcome
PSA	Prostate-specific antigen
RLT	Radioligand therapy
rPFS	Radiographic progression-free survival
SSB	Single-strand break
